# Age at menarche and idiopathic pulmonary fibrosis: a two-sample mendelian randomization study

**DOI:** 10.1186/s12890-024-02936-8

**Published:** 2024-03-06

**Authors:** Jiaqi Cao, Yazhou Ma, Wei Zhao, Chunlai Feng

**Affiliations:** 1 Department of Respiratory and Critical Care Medicine, The Third Affiliated Hospital of Soochow University, Changzhou, 213003 Jiangsu, China; 2 Department of Neurology, Third Affiliated Hospital of Soochow University, Changzhou, 213003 Jiangsu, China

**Keywords:** Age at menarche, Endogenous estrogen, Mendelian randomization, Idiopathic pulmonary fibrosis, Causal association

## Abstract

**Background:**

Sex difference in the incidence rate of idiopathic pulmonary fibrosis (IPF) indicates that estrogen has a certain protective effect on the disease. Nevertheless, there is a dearth of study investigating the association between factors pertaining to endogenous estrogen exposure level, such as age at menarche (AAM) in women, and IPF. Our study intended to employ Mendelian randomization (MR) method to elucidate the causal association between AAM and IPF.

**Methods:**

Our study utilized AAM as a measure of endogenous estrogen exposure and investigated its causal effect on the risk of IPF through MR. We employed the inverse variance weighted (IVW) method to assess the causal relationship between AAM and IPF risk, with supplementary analyses conducted using the weighted median estimator (WME) and MR-Egger method. Several sensitivity analyses were performed to assess the dependability of MR estimates.

**Results:**

A total of 9 selected single nucleotide polymorphisms (SNPs) significantly associated with AAM were selected as instrumental variables. The IVW method showed that genetically later AAM was associated with an increased risk of IPF (odds ratio [OR] = 1.0014, 95%confidence interval [CI] = 1.0005–1.0023, *p* = 0.001). The median weighting method and the MR-Egger method obtained similar estimates, and no heterogeneity or pleiotropy was found, indicating that the results were robust.

**Conclusions:**

Our MR study suggested a causal relationship between a later onset of menarche and a heightened susceptibility to IPF.

## Introduction

Idiopathic pulmonary fibrosis (IPF) is a type of interstitial lung disease characterized by chronic progressive pulmonary fibrosis accompanied by irreversible reduction of lung function [1]. The main pathological changes of IPF include epithelial cell damage, epithelial mesenchymal transition (EMT), deposition of extracellular matrix (ECM) proteins, and proliferation and differentiation of fibroblasts [2]. In recent years, the incidence of IPF has been increasing globally [3]. Once diagnosed with this disease, the survival period is short, the mortality rate is high, and the prognosis is poor [4]. Therefore, early identification and prevention for this disease are necessary. The incidence of IPF is higher in males compared to females, and this gender difference may be partly attributed to the differential exposure to sex hormones, particularly estrogen [5, 6].

The role of estrogen in IPF is still controversial. Some studies suggested that estrogen played a protective role in the development of IPF [7, 8]. However, an animal experiment found that, compared with males, female rats showed a higher mortality rate and more severe fibrosis [9]. Menarche is a milestone event in the sexual development of adolescent girls and is closely related to the level of endogenous estrogen exposure. Current studies on the relationship between age at menarche (AAM) and lung diseases mainly focus on asthma and lung cancer [10]. These studies showed that early menarche may lead to lower lung function in adult women and increased risk of asthma [11, 12], while later initiation of menstruation was associated with a reduced risk of developing lung cancer [13]. However, there are currently no clinical studies investigating the causal relationship between IPF and AAM. This may be due to the fact that surveys on respiratory diseases and AAM have primarily been conducted in younger cohorts, whereas IPF is predominately an elderly disease prone to recall bias during surveys of their AAM. Therefore, further research is needed to explore the relationship between AAM and IPF.

Mendelian randomization (MR) is a method that uses genetic variations as instrumental variables to investigate the potential causal relationship between exposure and outcome. MR is based on three assumptions. Firstly, genetic variations must be strongly associated with exposure. Secondly, genetic variations should not be correlated with any known confounding factors. Lastly, genetic variations should only affect the outcome through exposure [14]. Since alleles are randomly assigned at conception, they are not associated with confounding factors, and diseases cannot affect genetic variations, consequently, MR can also avoid reverse causal problems. We used AAM as a substitute index for endogenous estrogen exposure [15], selected single nucleotide polymorphisms (SNPs) that exhibited a strong correlation with AAM as instrumental variables, with IPF as the outcome, the method of two-sample MR was employed to analyze the causal association.

## Methods and materials

### Data source

The menarche age data were obtained from the MRbase database.

(https://gwas.mrcieu.ac.uk/), the genome-wide association study (GWAS) were derived from the Within Family Consortium, which included a total of 29,346 participants [16]. AAM is treated as a continuous measure in years, which is usually asked directly of study participants. As for the IPF data, we utilized a recent GWAS data as an outcome dataset, including 1369 patients with IPF and 435,866 controls [17]. The entire population is European. All the GWAS datasets can be obtained from IEU open GWAS Project (https://gwas.mrcieu.ac.uk/, Age at menarche GWAS ID: ieu - b − 4822. IPF GWAS ID: ebi-a-GCST90018120).

### Instrumental variable filtering

In the exposure database, we initially conducted a screening of SNPs using a genome-wide significance threshold of *p* < 5*10^-8. Subsequently, we eliminated SNPs that exhibited linkage disequilibrium (LD) with a threshold of R^2 < 0.001. The strength of the instrumental variables was assessed using the F-statistic. An instrumental variable is considered strong if the F-statistic > 10 [18]. All of the SNPs we utilized in our analysis had F statistics ranging from 55 to 78, well above the threshold of F-statistic > 10. This suggests that the existence of weaker instrumental variables is unlikely. For SNPs not found in the outcome summary data, we employed the online website tool LD-Link (http://ldlink.nih.gov) to identify proxy SNPs with a high degree of LD (r^2 > 0.8) [19]. Ultimately, a total of 9 SNPs were utilized for the MR analysis (Table 1).

**Table 1:** Characteristics of SNPs used as instrumental variables

SNP	EA	OA	EAF	Beta	SE	p-value	F
rs10978435	C	T	0.315574	-0.0898	0.0144	4.13E-10	69
rs16924631	C	G	0.13839	-0.0904	0.0164	3.67E-08	61
rs2090409	A	C	0.314781	-0.0967	0.013	1.05E-13	77
rs2362643	A	G	0.668282	-0.0874	0.0134	7.39E-11	75
rs314268	A	G	0.661809	-0.1401	0.0128	5.09E-28	78
rs543874	G	A	0.206329	-0.0792	0.0144	3.80E-08	69
rs7114175	T	A	0.499688	0.0746	0.0128	6.26E-09	78
rs72887143	T	A	0.16602	0.0879	0.0159	3.55E-08	63
rs79627842	C	T	0.127606	-0.1301	0.0182	8.23E-13	55

EA: effect allele; OA: other allele; EAF: effect allele frequency; SE, standard error; F, F-statistic

### Statistical analysis

We employed the inverse variance-weighted (IVW) method as our primary analysis approach, which is known to yield accurate results under the assumption that all instrumental variables meet the necessary assumptions [20]. Additionally, we utilized the weighted median estimator (WME) [21] and the MR-Egger method [22]. The WME method can provide relatively stable results when at least 50% of the instrumental variables satisfy the assumptions. Similarly, in the absence of heterogeneity, the MR-Egger method can yield relatively robust results. However, it is worth noting that the WME and MR-Egger methods exhibit slightly lower statistical efficiency, and thus, we employed them as supplementary analyses. To assess the heterogeneity among instrumental variables, we employed the Cochrane Q test [23]. Furthermore, we employed the MR-Egger intercept test [22] to evaluate whether the instrumental variables exhibit directional pleiotropy. A significant deviation of the intercept from zero suggests the presence of directional pleiotropy among the instrumental variables. Moreover, we employed the Mendelian Randomization Pleiotropy RESidual Sum and Outlier(MRPRESSO) method [24] to identify the outliers that may introduce horizontal pleiotropy. If outliers were detected, the corresponding SNPs were removed from the analysis. Additionally, we employed the leave-one-out method, which evaluates the remaining SNPs’ estimates of the results by deleting each SNP one by one, to evaluate the impact of individual SNPs on the results. All statistical analyses were conducted using the TwosampleMR package [25] and MRPRESSO package [24] in the R (version 4.1.2).

## Result

**Fig. 1:**
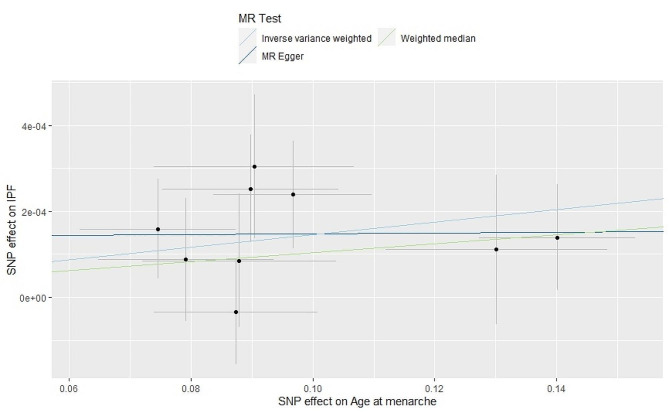
Scatter plot for the causal effect of AAM on IPF risk

The results obtained through the IVW method indicate a positive correlation between the AAM and the increased risk of IPF (odds ratio [OR] = 1.0014, 95% confidence interval [CI] = 1.0005∼1.0023, *p* = 0.001). Similar estimates were obtained through the WME method (OR = 1.001, 95% CI = 1.000∼1.002, *p* = 0.093) and the MR-Egger method (OR = 1.000, 95% CI = 0.996∼1.004, *p* = 0.967) (Fig. 1). However, these supplementary methods have lower testing efficiency, particularly the MR-Egger method, resulting in larger confidence intervals.

**Fig. 2:**
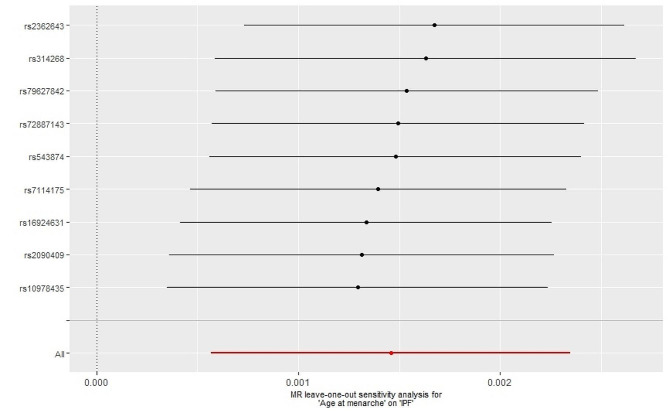
Leave-one-out analysis of the effect of Age at menarche on IPF

The Cochran’s Q test results indicated that there was no heterogeneity observed among the SNPs (Q = 98.46, *p* = 0.15). Additionally, the MR-Egger intercept test revealed an intercept value of 0.012 and a p-value of 0.39, suggesting the absence of directional pleiotropy in the results. Furthermore, there was no outlier displayed by MRPRESSO method, which indicated that the results were not influenced by abnormal SNPs and exhibited pleiotropy. The leave-one-out method analysis demonstrated that none of the SNPs had a significant impact on the results (Fig. 2). Consequently, our findings can be considered robust.

## Discussion

Our study found that a later onset of menarche is associated with an increased risk of IPF. We used AAM as a surrogate of the level of exposure to endogenous estrogen. A later AAM means a lower cumulative level of exposure to endogenous estrogen, further supporting the hypothesis that endogenous estrogen plays a protective role in IPF.

IPF exhibits a higher prevalence in males, with males experiencing an earlier age of onset and a poorer overall survival rate compared to females. Consequently, female gender is considered as a protective factor against the development of this disease [26]. Males have less accumulation of endogenous estrogen throughout their lifetime in comparison to females. In the female population, a later AAM corresponds to reduced exposure to endogenous estrogen. Our result suggested that a delayed AAM was associated with an increased risk of developing IPF, thereby supporting the protective role of female gender and estrogen in the incidence of IPF. However, there is a lack of clinical studies that have examined the impact of gender-related factors, such as AAM and estrogen levels, on the incidence and prognosis of IPF. Therefore, the result of our study has important guiding significance for future clinical investigations in this area.

The effect of AAM on the risk of IPF can be mediated by estrogen. Research has shown that estrogen can exert anti-inflammatory effects by decreasing tumor necrosis factor-α production, interferon-γ expression and natural killer cell activity [27]. Inflammatory response is also an important mechanism of IPF [28], therefore estrogen may play a protective role in IPF through anti-inflammatory effects. EMT is an important pathological process in IPF [29]. Transforming growth factor-β (TGF-β)/Smad cascade is a key fibrotic axis that drives EMT by increasing the ECM synthesis and collagen deposition. Andugulapati SB et al. found that, phytoestrogen Biochanin-A mitigated the development and progression of pulmonary fibrosis by modulating the TGF-β/Smad3 pathway and ameliorating the fibrotic cascade of events [30]. Furthermore, estradiol could specifically down-regulate the expression of chloride intracellular channel protein 3 and retinol binding protein 7 genes related to the pathogenesis of pulmonary fibrosis in human bronchial epithelial cells, and affect the pathways associated with pulmonary fibrosis [7]. Additionally, 2-methoxy estradiol, a metabolite of estradiol in the human body, has been found to inhibit the growth of human pulmonary artery smooth muscle cells and human lung fibroblasts in a concentration-dependent manner. It also exhibited strong anti-inflammatory, anti-fibrosis, and anti-vascular remodeling effects in models of pulmonary fibrosis and hypertension [31].

Several studies conducted on mice with IPF also support that estrogen may have a protective role in IPF. Female mice displayed relatively mild pulmonary fibrosis changes and longer survival compared to male mice [32, 33]. Oophorectomy in female mice gave rise to significant thickening of airway smooth muscle and promoted the progression of airway fibrosis, which could be reversed by estrogen replacement therapy [34]. However, in a study targeting rats, the opposite phenomenon was observed: female rats experienced more severe bleomycin induced pulmonary fibrosis than age matched male rats, while female rats experienced reduced pulmonary fibrosis after oophorectomy, and estrogen replacement therapy exacerbated pulmonary fibrosis [9]. We speculate that this phenomenon may be related to the complex mechanism of estrogen’s action in the lungs [10]. In addition, SolopovP et al. discovered that the supplementation of phytoestrogens acting by stimulating estrogen receptors could improve lung function impairment in murine models of hydrochloric acid-induced pulmonary fibrosis [8]. The findings of these studies are mutually confirmed with the result of our study to some extent. Early identification and intervention of IPF patients with late menarche are needed to improve the prognosis of the disease and prolong survival. In the future, it is still necessary to further explore the specific mechanism of estrogen’s role in IPF, so as to develop new treatments.

This study represents the first application of MR to investigate the potential causal relationship between AAM and IPF. MR studies offer advantages over traditional observational studies by reducing the risk of confounding. In this study, we utilized recently published large-scale GWAS data, which provide increased statistical power due to larger sample sizes. Furthermore, we conducted multiple sensitivity analyses to assess the robustness of our findings. Consequently, the results of our study hold significant reference value for informing future clinical research. However, there are several limitations to consider. Firstly, we used GWAS data on menarche from the Within Family Consortium, which includes both men and women, the lack of sex-specific data from the public database weakens the causal relationship between AAM and IPF in our study, making our results more conservative. Secondly, all participants in our study were of European descent, thus limiting the generalizability of our findings to other ethnicities. Further cross-ethnic studies, if corresponding GWAS data are available, are necessary to address this limitation. Thirdly, we did not use age of menopause as an exposure because after screening the GWAS instrumental variables for menopausal age, only three SNPs were obtained as instrumental variables. We believe that the instrumental variables are too few to serve as a genetic proxy for age of menopause. In the future, when there are larger scale GWAS studies on age of menopause and more instrumental variables for age of menopause can be obtained, MR analysis can be conducted on the relationship between age of menopause and IPF. Lastly, it is important to note that MR assumes a linear correlation between AAM and IPF, although the actual relationship may not necessarily follow this pattern.

## Conclusion

Our research has presented the finding that suggests a correlation between a delayed AAM and an elevated susceptibility to developing IPF, possibly attributed to changes in endogenous estrogen levels. Further studies are necessary to validate this hypothesis and elucidate the precise molecular mechanisms underlying this relationship.

## Data Availability

All data generated or analysed in our study are included in this published article.

## References

[1] Barratt SL, Creamer A, Hayton C (2018). Idiopathic pulmonary fibrosis (ipf): an overview. J CLIN MED.

[2] Confalonieri P, Volpe MC, Jacob J (2022). Regeneration or repair? The role of alveolar epithelial cells in the pathogenesis of idiopathic pulmonary fibrosis (ipf). Cells (Basel Switzerland).

[3] Hutchinson J, Fogarty A, Hubbard R (2015). Global incidence and mortality of idiopathic pulmonary fibrosis: a systematic review. EUR RESPIR J.

[4] Moss BJ, Ryter SW, Rosas IO. Pathogenic Mechanisms Underlying Idiopathic Pulmonary Fibrosis. ANNU REV PATHOL-MECH. 2022; 17:515–46. 10.1146/annurev-pathol-042320-030240. 10.1146/annurev-pathol-042320-030240 34813355

[5] Raghu G, Weycker D, Edelsberg J (2006). Incidence and prevalence of idiopathic pulmonary fibrosis. AM J RESP CRIT CARE.

[6] Sathish V, Martin YN, Prakash YS. Sex Steroid Signaling: Implications for Lung Diseases., Pharmacology. & therapeutics (Oxford). 2015; 150:94–108. 10.1016/j.pharmthera.2015.01.007. 10.1016/j.pharmthera.2015.01.007 PMC4523383 25595323

[7] Smith LC, Moreno S, Robertson L (2018). Transforming growth factor Beta1 targets estrogen receptor signaling in bronchial epithelial cells. RESP RES.

[8] Solopov P, Colunga Biancatelli RML, Dimitropoulou C (2021). Dietary Phytoestrogens Ameliorate Hydrochloric Acid-Induced Chronic Lung Injury and Pulmonary Fibrosis in mice. NUTRIENTS.

[9] Gharaee-Kermani M, Hatano K, Nozaki Y (2005). Gender-based differences in Bleomycin-Induced Pulmonary Fibrosis. AM J PATHOL.

[10] Macsali F, Svanes C, Bj rge L (2012). Respiratory health in women: from Menarche to Menopause. EXPERT REV RESP MED.

[11] Macsali F, Real FG, Plana E (2011). Early Age at Menarche, lung function, and adult asthma. AM J RESP CRIT CARE.

[12] Minelli C, van der Plaat DA, Leynaert B (2018). Age at Puberty and Risk of Asthma: a mendelian randomisation study. PLOS MED.

[13] Denos M, Sun Y, Jiang L (2023). Age at Menarche, Age at Natural Menopause, and risk of lung and colorectal cancers: a mendelian randomization study. J ENDOCR SOC.

[14] Davies NM, Holmes MV, Davey SG (2018). Reading mendelian randomisation studies: a Guide, Glossary, and Checklist for clinicians. BMJ-BRIT MED J.

[15] Neumeyer S, Banbury BL, Arndt V (2018). Mendelian randomisation study of age at Menarche and Age at Menopause and the risk of Colorectal Cancer. BRIT J CANCER.

[16] Howe LJ, Nivard MG, Morris TT (2022). Within-sibship genome-wide Association analyses decrease Bias in estimates of Direct Genetic effects. NAT GENET.

[17] Duckworth A, Gibbons MA, Allen RJ (2021). Telomere length and risk of Idiopathic Pulmonary Fibrosis and Chronic Obstructive Pulmonary Disease: a mendelian randomisation study. LANCET RESP MED.

[18] Pierce BL, Ahsan H, Vanderweele TJ (2011). Power and Instrument Strength requirements for mendelian randomization studies using multiple genetic variants. INT J EPIDEMIOL.

[19] Machiela MJ, Chanock SJ, Ldlink (2015). A web-based application for Exploring Population-Specific Haplotype structure and linking correlated alleles of possible functional variants. Bioinformatics.

[20] Bowden J, Del GMF, Minelli C, et al. A Framework for the Investigation of Pleiotropy in Two-Sample Summary Data Mendelian Randomization. STAT MED. 2017; 36(11):1783-1802. 10.1002/sim.7221 10.1002/sim.7221 PMC5434863 28114746

[21] Bowden J, Davey SG, Haycock PC (2016). Consistent estimation in mendelian randomization with some Invalid instruments using a weighted median estimator. GENET EPIDEMIOL.

[22] Burgess S, Thompson SG (2017). Interpreting findings from mendelian randomization using the Mr-Egger Method. EUR J EPIDEMIOL.

[23] Egger M, Smith GD, Phillips AN, BMJ-BRIT MED (1997). J.

[24] Verbanck M, Chen CY, Neale B (2018). Detection of widespread horizontal pleiotropy in Causal relationships inferred from mendelian randomization between Complex traits and diseases. NAT GENET.

[25] Hemani G, Zheng J, Elsworth B, et al. The Mr-Base platform supports systematic causal inference across the human phenome. ELIFE. 2018;7. 10.7554/eLife.34408. 10.7554/eLife.34408 PMC5976434 29846171

[26] Han MK, Murray S, Fell CD (2008). Sex differences in physiological progression of idiopathic pulmonary fibrosis. Eur Respir J.

[27] Balda ara RPDC, Silva I (2017). Association between Asthma and female sex hormones. S?o Paulo Med J.

[28] Jia Q, Lei Y, Chen S (2023). Circulating inflammatory cytokines and risk of idiopathic pulmonary fibrosis: a mendelian randomization study. BMC PULM MED.

[29] Todd NW, Luzina IG, Atamas SP (2012). Molecular and Cellular mechanisms of Pulmonary Fibrosis. Fibrogenesis Tissue Repair.

[30] Andugulapati SB, Gourishetti K, Tirunavalli SK (2020). Biochanin-a ameliorates pulmonary fibrosis by suppressing the tgf-Beta mediated emt, myofibroblasts differentiation and Collagen Deposition in in Vitro and in vivo systems. Phytomedicine.

[31] Tofovic SP, Zhang X, Jackson EK (2009). 2-Methoxyestradiol attenuates Bleomycin-Induced Pulmonary hypertension and fibrosis in estrogen-deficient rats. VASC PHARMACOL.

[32] Solopov P, Colunga BR, Dimitropoulou C, et al. Sex-related differences in Murine models of Chemically Induced Pulmonary Fibrosis. INT J MOL SCI. 2021;22(11). 10.3390/ijms22115909. 10.3390/ijms22115909 PMC8198091 34072833

[33] Redente EF, Jacobsen KM, Solomon JJ (2011). Age and sex dimorphisms contribute to the Severity of Bleomycin-Induced Lung Injury and Fibrosis. AM J PHYSIOL-LUNG C.

[34] Lekgabe ED, Royce SG, Hewitson TD (2006). The effects of Relaxin and Estrogen Deficiency on Collagen Deposition and hypertrophy of nonreproductive organs. Endocrinology.

